# Alternative splicing landscape of the neural transcriptome in a cytoplasmic-predominant Pten expression murine model of autism-like Behavior

**DOI:** 10.1038/s41398-020-01068-x

**Published:** 2020-11-06

**Authors:** Stetson Thacker, Marilyn Sefyi, Charis Eng

**Affiliations:** 1 Genomic Medicine Institute, Lerner Research Institute, Cleveland Clinic, Cleveland, OH 44195 USA; 2 Cleveland Clinic Lerner College of Medicine, Cleveland, OH 44195 USA; 3 Taussig Cancer Institute, Cleveland Clinic, Cleveland, OH 44195 USA; 4 Department of Genetics and Genome Sciences, Case Western Reserve University School of Medicine, Cleveland, OH 44106 USA; 5 Germline High Risk Cancer Focus Group, Comprehensive Cancer Center, Case Western Reserve University School of Medicine, Cleveland, OH 44106 USA

**Keywords:** Autism spectrum disorders, Genomics

## Abstract

Alternative splicing (AS) is a posttranscriptional mechanism regulating gene expression that complex organisms utilize to expand proteome diversity from a comparatively limited set of genes. Recent research has increasingly associated AS with increased functional complexity in the central nervous systems in higher order mammals. This work has heavily implicated aberrant AS in several neurocognitive and neurodevelopmental disorders, including autism. Due to the strong genetic association between germline *PTEN* mutations and autism spectrum disorder (ASD), we hypothesized that germline *PTEN* mutations would alter AS patterns, contributing to the pathophysiology of ASD. In a murine model of constitutional mislocalization of Pten, recapitulating an autism-like phenotype, we found significant changes in AS patterns across the neural transcriptome by analyzing RNA-sequencing data with the program rMATS. A few hundred significant alternative splicing events (ASEs) that differentiate each m3m4 genotype were identified. These ASEs occur in genes enriched in PTEN signaling, inositol metabolism, and several other pathways relevant to the pathophysiology of ASD. In addition, we identified expression changes in several splicing factors known to be enriched in the nervous system. For instance, the master regulator of microexons, Srrm4, has decreased expression, and consequently, we found decreased inclusion of microexons in the *Pten*^*m3m4/m3m4*^ cortex (~10% decrease). We also demonstrated that the m3m4 mutation disrupts the interaction between Pten and U2af2, a member of the spliceosome. In sum, our observations point to germline *Pten* disruption changing the landscape of alternative splicing in the brain, and these changes may be relevant to the pathogenesis and/or maintenance of PTEN-ASD phenotypes.

## Introduction

Metazoans utilize alternative splicing (AS), nominally a posttranscriptional mechanism (though the process occurs co-transcriptionally) by which various exonic segments are excised from intronic segments of the gene-encoded RNA message and then conjoined combinatorically to form a diverse set of transcriptional messages, to exponentially expand the coding potential of the genome^1–3^. Thus, AS is primarily responsible for generating the extensive proteomic diversity of complex organisms, explaining the correlation between AS and organismal complexity, a correlation that is thought to be important to and illuminating of the functions of the central nervous system (CNS) in humans^4^. Pathological AS patterns have been associated with a host of neurodevelopmental and neurodegenerative disorders, including idiopathic autism spectrum disorder (ASD)^5–7^.

Germline mutation in *PTEN*, canonically a tumor suppressor gene, is the molecular criterion for the diagnosis of PTEN Hamartoma Tumor Syndrome (PHTS), an autosomal dominant cancer predisposition syndrome where individuals present variably. Macrocephaly, benign and malignant neoplasia, and neurodevelopmental phenotypes (i.e., developmental delay and ASD) are the common clinical manifestation of PHTS that are of ostensibly great import to patients and clinicians^8–10^. Moreover, *PTEN* is one of the most strongly associated autism risk genes with ~23% of individuals with germline mutations receiving an ASD diagnosis and an even greater percentage with developmental delay and other neurological phenotypes^9,11,12^.

The *Pten*^*m3m4*^ mouse is a model of cytoplasmic-predominant Pten expression, resulting from five nucleotide substitutions distributed across the third and fourth nuclear localization-like signals of *Pten* in exon 7^13^. The *Pten*^*m3m4*^ mouse presents with macrocephaly secondary to megencephaly and an autism-like behavioral phenotype, specifically a sex-specific increase in social motivation with intact spatial and social recognition memory and motor learning^13^. Importantly, the neural transcriptome of these mice reveals differentially expressed genes similar to those associated with idiopathic autism^14^. These organismal, molecular, and cellular phenotypes characterize the *Pten*^*m3m4*^ mouse as a suitable model of idiopathic autism. For example, there are numerous neuronal and glial phenotypes, including hypertrophy of neuronal soma, astrogliosis, microglial activation, and white matter abnormalities^13,15^. Additional study found that the *Pten*^*m3m4*^ oligodendrocytes mature precociously, leading to more NG2-positive cells, and myelinate aggressively but improperly^16^. Furthermore, we discovered cell autonomous activation of microglial that participate in increased synaptic pruning due to inherently increased phagocytic activity and increased “eat me” signals from *Pten*^*m3m4*^ neurons^17^. Moreover, RNA-sequencing found that many known ASD-risk genes annotated by the Simons Foundation Autism Research Initiative are differentially expressed in the brain and cortex of the *Pten*^*m3m4*^ mouse. Generally, the *Pten*^*m3m4*^ neural transcriptome mimicked that of idiopathic ASD, demonstrating an increase in expression of markers of inflammation and a decrease in markers of synaptic transmission^14^. Considering the association of *PTEN* with ASD and even more recently with the regulation AS^18^, we hypothesized that the neural transcriptome of the *Pten*^*m3m4*^ mouse will show differential patterns of AS and that these patterns will associate with pathophysiological hallmarks of ASD.

## Materials and methods

### Animals

Generation and characterization of the *Pten*^*m3m4*^ mouse on a CD1 background has been described previously^13^. The *Pten*^*m3m4*^ mutation is located within exon 7 of *Pten* and consists of five nucleotide substitution mutations, resulting in four nonsynonymous and one synonymous amino acid changes in the third and fourth putative nuclear localization-like sequences of *Pten*^13,19^. Genotyping was performed on genomic DNA from clipped toes per the Jackson Laboratory protocol using modified PCR primers. Wild-type allele primers: mPten-F5, 5′-TGGCAGACTCTTCATTTCTGTGGC-3′, and mPten-R6, 5′-ACTTCTTCACAACCACTTCTTTCAAC-3′. Mutant allele primers are mPten-F3, 5′-TACCCGGTAGAATTTCGACGACCT-3′, and mPten-R6, 5′-ACTTCTTCACAACCACTTCTTTCAAC-3′. Mice were maintained on a 14:10 light:dark cycle with access to food and water *ad libitum*. The room temperature was maintained between 18 and 26 °C. Animals were euthanized via CO_2_ asphyxiation followed by cervical dislocation. All experiments were conducted under protocols approved by the Institutional Animal Care and Use Committee at Cleveland Clinic.

### Transcriptomic data analysis

The cortical transcriptome of 6-week-old *Pten*^*m3m4*^ mice (GSE59318) shares significant similarity with the genes associated witAS h idiopathic autism^14^. In our study, we utilize results from the published TopHat2-Cuffdiff pipeline and construct a new pipeline to re-process the raw RNA-sequencing data (i.e., fastq files) for AS analysis. In the new pipeline, we performed our alignment with Spliced Transcripts Alignment to a Reference (STAR version 2.7). The resulting bam files were then used as input for the analysis of AS. To confirm that the differential expression (DE) profile was concordant with two different alignments, we processed transcript per million data from bam files to assess DE across *Pten*^*m3m4*^ genotypes with NOISeq (3.10). For pairwise genotype comparisons of DE, the second listed genotype is the denominator while the first listed genotype is the numerator for fold change calculations (e.g., MUT vs. WT → MUT/WT = Fold Change). The STAR-NOISeq pipeline DE data showed extensive similarity to TopHat2-Cuffdiff pipeline, confirming to us that the STAR alignment did not alter the biological observation of our previous study and was suitable to move forward with AS analysis.

### Analysis of alternative splicing patterns from RNA-sequencing data

We took two approaches to analyze AS in the *Pten*^*m3m4*^ mouse brain. The first relied on our previously published Cuffdiff pipeline that returned a differential transcript expression analysis that was unpublished in our study of the *Pten*^*m3m4*^ neural transcriptome, including both the 2-week (P14) and 6-week (P40) time points^14^. However, to obtain exon-level resolution, we performed Multivariate Analysis of Transcript Splicing for replicates (rMATS version 4.0.2) to robustly and flexibly detect differential alternative splicing events (ASEs). We applied rMATS 4.0.2 on bam files from all genotypes of *Pten*^*m3m4*^ neural transcriptome and then subsequently filtered the output by significance (*p*), false discovery rate (FDR), and change in PSI (delta PSI or ΔΨ) values for various subsequent analyses. All ΔΨ values for ASEs from pairwise comparisons are reported with the second listed genotype as the PSI value subtracted from the PSI value of the first listed PSI value (e.g., MUT vs. WT → MUT – WT = ΔΨ). All of our scripts are located on our GitHub page (https://github.com/Thackes/PTEN-AlternativeSplicing).

### Gene enrichment and pathway analysis

We performed a “Core Analysis” using Ingenuity Pathway Analysis (IPA) software on the list of genes that harbor ASEs meeting the significance (*p*), FDR, and delta PSI (i.e., ΔΨ) thresholds (Qiagen, Redwood City, California). The basis for the “Core Analysis” were the experimental *p* values. Delta PSI values were not included in the analysis so that IPA would not treat these values as directional changes in gene expression. In the IPA analysis settings, we generally retained the default parameters except that we limited our species’ background to mouse and included only experimentally observed relationships. The top canonical pathways, diseases and bio functions, and networks were analyzed and reported. Special attention was given to highly significant pathways and networks relevant to neurodevelopmental phenotypes. Summaries of the entire IPA analyses are available on our GitHub page.

### Srrm4 expression and microexon analysis

We quantified Srrm4 expression using SYBR qRT-PCR with the following primers: Srrm4-Forward, 5′-CAGAGAGTCAAGAGGGTTTCAG-3 and Srrm4-Reverse, 5′-GTGGATGGGACCAAACTAGAC-3′. Following Srrm4 expression analysis, we assessed microexon splicing with RT-PCR, running the small fragments in high-grade 3% agarose gels. The microexon primers are described in Supplementary Table 1 and the RT-PCR protocol was carried out as in^20^.

### Rapid Amplification of cDNA Ends (RACE) sequencing

We used and adapted the *Nature Method* for Rapid Amplification of cDNA Ends (RACE) sequencing protocol^21^ for a 3′ approach. Several gene targets of interest as identified by the most differentially expressed transcripts in the Cuffdiff analysis^14^ were subject to RACE sequencing, including *Gfap*. The *Gfap* RACE primer we used was 5′-GATTACGCCAAGCTTCGCATTTGCCGCTCTAGGGACTC-3′.

### Immunoprecipitation and Western blot analysis

The cortex was isolated, snap-frozen in liquid nitrogen, and stored at −80 °C. To lyse, we thawed the tissue on ice and then lysed in RIPA buffer (10-mM Tris-Cl [pH 8], 1-mM EDTA, 0.5-mM EGTA, 1% Triton X-100, 0.1% sodium deoxycholate, 0.1% SDS, 140-mM NaCl, and before use add 1-mM PMSF) with phosphatase inhibitor #2 (Sigma, St. Louis, Missouri, #P5726-5ML), phosphatase inhibitor #3 (Sigma, #P0044-5ML) and protease inhibitor (Sigma, #P8345-5ML). These lysates were quantified for total protein content (mg/mL) using BCA assays, and then the lysates were diluted to equal concentrations. Next, 15 μg of protein per sample was loaded on a 4–12% gradient polyacrylamide gel and separated by standard SDS-PAGE. The separated proteins were transferred to a nitrocellulose membrane, and the membrane was blocked for 4 h or overnight in 2.5% milk diluted in Tris-buffered saline, containing 0.2% Tween-20 (TBST) at 4 °C. Membranes were then washed with TBST and incubated with experiment specific primary antibodies diluted in TBST overnight at 4 °C. The following antibodies were used: PTEN (1:2000, #ABM-2025, Cascade Bioscience, Winchester, Massachusetts) and U2AF2 (1:1000, #ab37530, Abcam, Cambridge, Massachusetts). Following incubation, we removed the primary antibody and performed three 10 min washes with TBST. Blots were probed with goat anti-mouse secondary antibody IRDye800CW (1:20,000, #213965, LI-COR, Lincoln, Nebraska) or Goat Anti-Rabbit IRDye680 (LI-COR, #213971) diluted in TBST, for 2 h at room temperature. The membranes were washed for 10 min three times each in TBST and then imaged using the Odyssey CLx imaging system (LI-COR).

### Statistical analysis

We analyzed normally distributed data using a one-way analysis of variance (ANOVA) or Student’s *t-*test, where appropriate (Graph Pad Prism 7). After performing a one-way ANOVA, we performed a post hoc Tukey-Kramer analysis (F). When data were not normally distributed, we performed non-parametric inference tests such as Mann-Whitney U and Kruskall–Wallis tests (H), where appropriate (Graph Pad Prism 7). *P* values that are less than 0.05 were considered statistically significant.

## Results

**Fig. 1:**
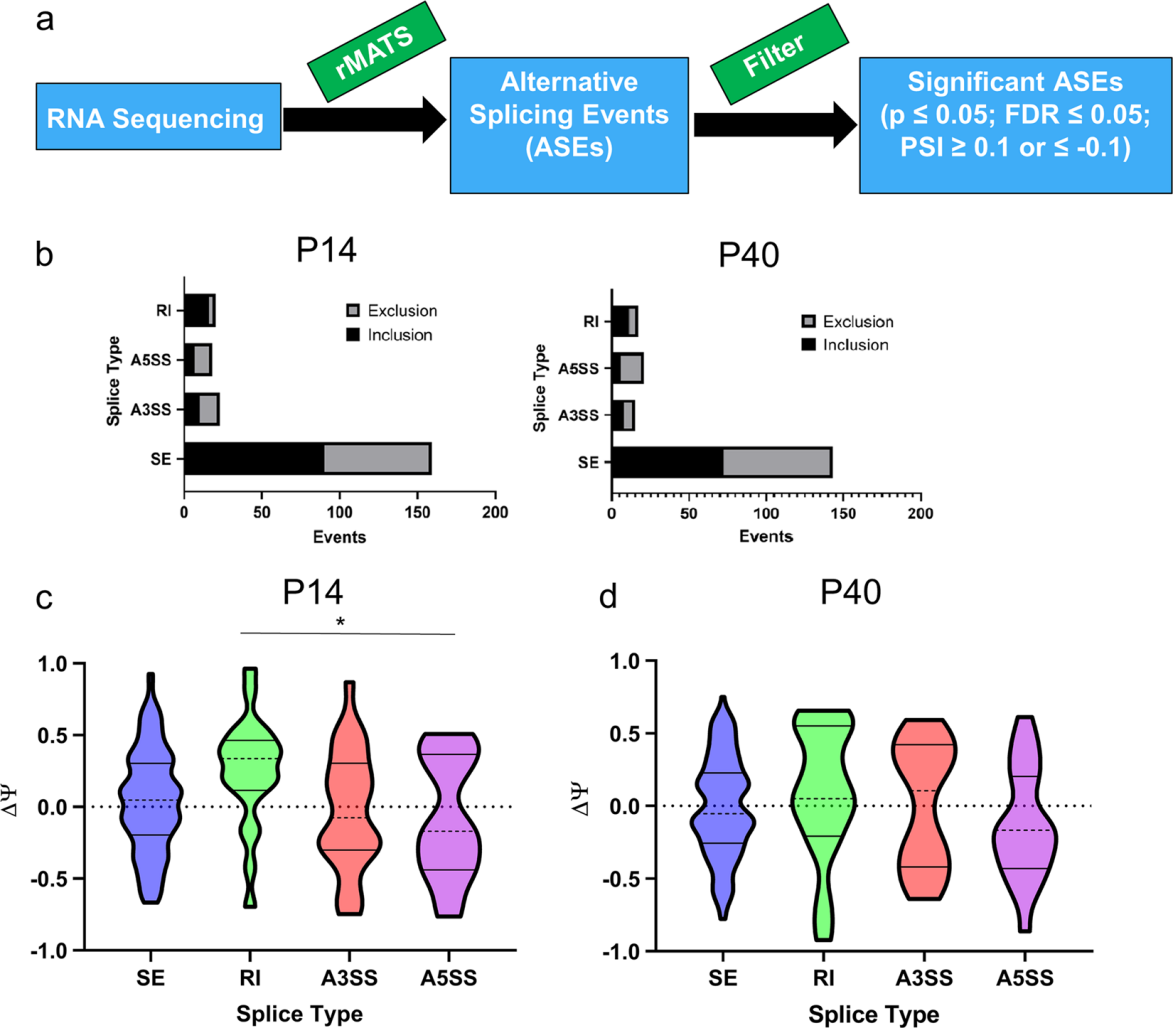
Global patterns of alternative splicing patterns in the *Pten*^*m3m4*^ mouse brain. **a** Schematic pipeline for analysis of alternative splicing patterns from RNA-sequencing data using rMATS (*N* = 3 for each genotype at each time point). **b** Summary of inclusion and exclusion events for MUT vs. WT comparison by event type for P14 and P40 time points. **c**, **d** ΔΨ distributions by event type for MUT vs. WT comparison for both P14 and P40 time points (*p < 0.05). SE exon skipping, RI retained intron, A3SS alternative 3′ splice site; A5SS alternative 5′ splice site.

In order to assess AS changes in the neural transcriptome of *Pten*^*m3m4*^ mice in the 2-week old (i.e., P14) hemibrain and 6-week old (i.e., P40) cortex, we deployed rMATS to detect and analyze ASEs across all genotypes at both time points on previously published RNA-sequencing data^14^. Subsequently, three comparisons were performed: wild-type versus homozygous mutant (MUT vs. WT), heterozygous versus wild-type mutant (HET vs. WT), and homozygous mutant versus heterozygous mutant (MUT vs. HET). All delta PSI (ΔΨ) values were calculated as consistent with the following example: MUT vs. WT → PSI MUT – PSI WT = Delta PSI. We considered ASEs to meet our threshold for subsequent analysis if they met three criteria: *p* ≤ 0.05, FDR ≤ 0.05, and ΔΨ ≤ −0.1 or ΔΨ ≥ 0.1. For some analyses, where noted, the PSI filter was omitted. A schematic illustrating our rMATS analysis is shown in Fig. 1A.

### Global splicing analysis of *Pten*^*m3m4*^ neural transcriptome

The rMATS analysis identified a modest number of ASEs, 220 and 194, passing our significance and false discovery criteria that differed between the homozygous mutant and wild type (MUT vs. WT) at both P14 and P40 time points, respectively (Fig. 1b; Supplementary Table 2). There were also similar numbers of ASEs identified in the other two comparisons at both time points (Supplementary Fig. 1a). This suggests each m3m4 allele introduces a significant effect on the landscape of AS in the neural transcriptome, making *Pten*^*w/wt*^, *Pten*^*wt/m3m4*^, and *Pten*^*m3m4/m3m4*^ brain equally distinct from one another.

In order to gain a greater understanding of the kinds of ASEs the rMATS analysis identified, we evaluated the changes by splicing event type: exon skipping (SE), intron retention (RI), alternative 3′ splice site (A3SS), and alternative 5′ splice site (A5SS). Due to the small number of mutually exclusive exon splicing events, we have recorded those data in the supplemental information for all comparisons (Supplementary Table 3). Furthermore, for the sake of simplicity and the greater biological interest in the MUT vs. WT comparison, the other splicing event type data are displayed in the supplemental information (Supplementary Fig. 1b). We thus focused on the MUT vs. WT splicing event data, where we found that SE was the most common type of ASE at both time points. At P14, we identified 90 inclusion (i.e., positive ΔΨ) and 69 exclusion (i.e., negative ΔΨ) SE events. At P40, we identified 72 inclusion and 71 exclusion events. For RI, we found 16 and 11 inclusion events and 4 and 6 exclusion events at P14 and P40, respectively. In addition, for A3SSs, we found 10 and 8 inclusion events and 13 and 7 exclusion events at P14 and P40, respectively. Finally, for A5SSs, we found 7 and 6 inclusion events and 11 and 15 exclusion events at P14 and P40, respectively (Fig. 1b). These data not only show that SE is the predominant event type, but also that generally the directionality (i.e., inclusion or exclusion) of ASEs, regardless of event type, are distributed roughly equally. There are a few important exceptions to this observation: SE events at P14, A3SS events at P40, and RI events at both time points.

Towards a comprehensive perspective on global splicing pattern in the *Pten*^*m3m4/m3m4*^ P14 hemibrain and P40 cortex, we evaluated the ΔΨ distributions for ASEs meeting our significance and FDR filters by event type. At P14, we found evidence that the ΔΨ distributions differ significantly by event type (*p* = 0.023). This trend is driven by the increase in RI in the homozygous mutant. There is significant evidence of an increase in ΔΨ for RI events vs. ΔΨ for A3SS events (*p* = 0.039), and a trend for an increase in ΔΨ for RI event vs. ΔΨ for A3SS events (*p* = 0.055) and SE events (*p* = 0.053), where the median ΔΨ is ~30% more towards the direction of inclusion than the next closest event type (34% for RI vs. 4.7% for SE). At P40, we found a similar trend towards an increase in RI, but the trend was not statistically significant (*p* = 0.16; Fig. 1c). These data illustrate that there is generally increased RI and decreased SE and other splice type events in the *Pten*^*m3m4/m3m4*^ brain.

### Enrichment and pathway analysis finds PTEN signaling and neurologically related genes affected by splicing changes

**Fig. 2:**
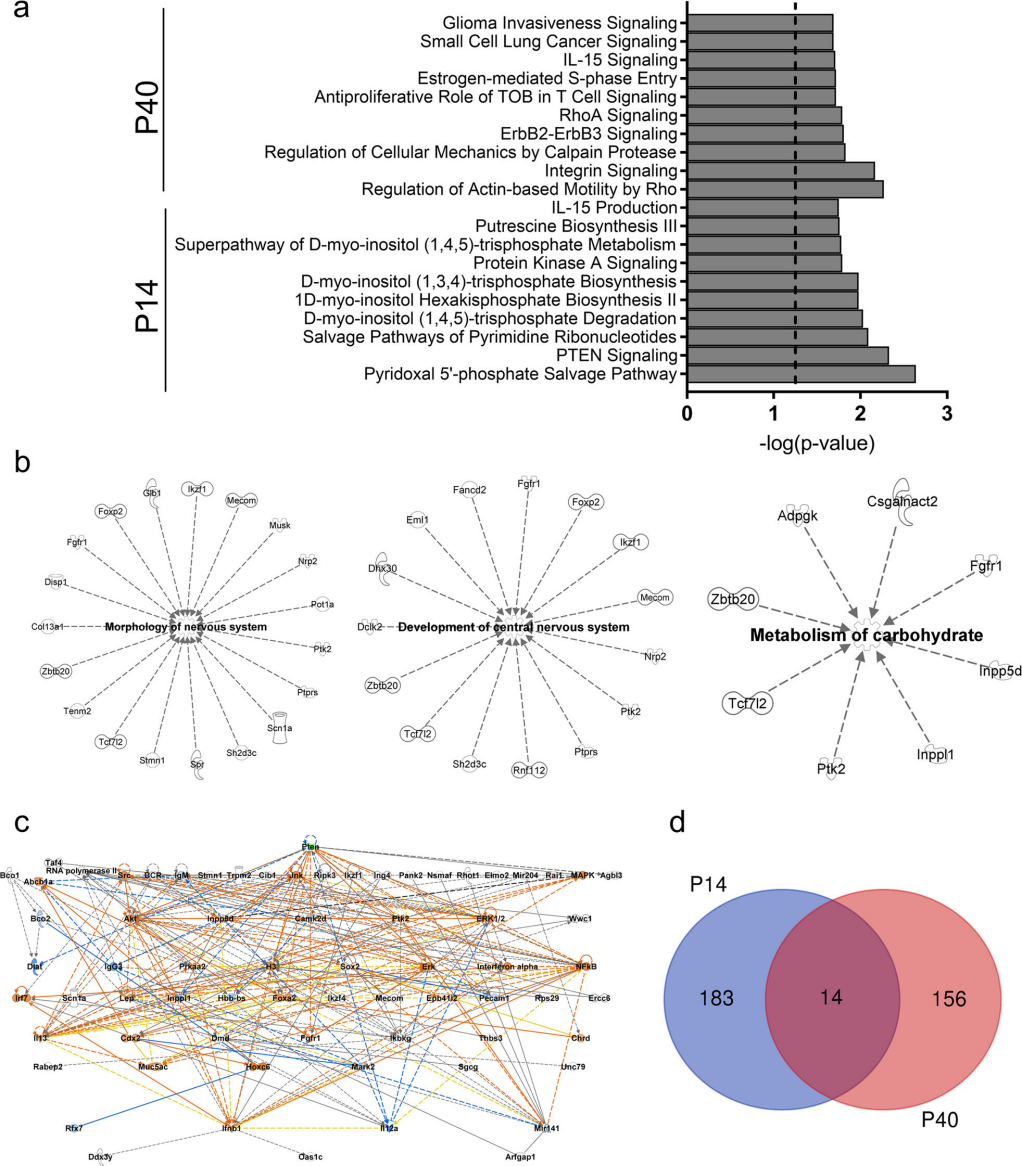
Pathways and networks enriched in the genes with ASEs *Pten*^*m3m4/m3m4*^ mouse brain. **a** Top ten canonical pathways identified by Ingenuity Pathway Analysis (IPA) for P14 and P40 time points. **b** PTEN phenotype relevant “disease and bio function” networks identified by IPA at P14. From right to left: morphology of nervous system, development of central nervous system, and metabolism of carbohydrate. **c** Merged top networks from P14 and P40 with PTEN overlay and biological predictions using the Molecule Activity Predictor (MAP) tool on IPA. Green indicates the decrease in Pten expression observed in models with the m3m4 mutation. Orange molecules are predicted to be activated, while blue molecules are predicted to be inhibited. Edges that are orange are predicted activated, while those that are blue are inhibited, gray are without directional prediction, and yellow are inconsistent with the activity state of the connected node. The network is organized hierarchically so that higher nodes have regulatory influence over lower nodes. **d** Venn diagram of intersecting and separate genes with significant ASEs at P14 and/or P40.

To gauge the putative functional consequences of the altered landscape of AS in the *Pten*^*m3m4/m3m4*^ brain, we performed a core analysis in IPA, which performs gene enrichment, pathway, and network analyses on the list of genes in which ASEs of interest occurred. At P14, the top ten most enriched canonical pathways include those related to phosphatidylinositol head group metabolism, PTEN signaling, and PKA signaling; whereas at P40, the top ten most enriched canonical pathways include those related to Rho and integrin signaling (Fig. 2A). Investigating “Diseases and Bio Function” networks at P14, which appears to be the more critical time period for ASEs relevant to Pten function, we found several enriched networks relevant to the phenotypes of the *Pten*^*m3m4*^ mouse: Morphology of Nervous System, Development of CNS, and Metabolism of Carbohydrate (Fig. 2b). These data illustrate the importance of the genes with ASEs to ASD-related phenotypes that are manifest in the *Pten*^*m3m4*^ mouse.

In addition, we evaluated networks of genes experiencing ASEs and closely related molecules that IPA identified as enriched. We took the top scoring networks (i.e., those involving the largest number of interrelated genes experiencing ASEs) from both time points and merged them. Then, using the Molecule Activity Predictor tool in IPA, we input a decrease in Pten expression, an observed phenotype in the *Pten*^*m3m4/m3m4*^ brain, and observed the predicted biological response throughout the merged P14 and P40 network (Fig. 2c). Based on the decrease Pten expression, IPA predicted the activation of numerous influential nodes: Src, Jnk, Akt, Erk1/2, and Mapk. The network is organized hierarchically so that molecules at higher tiers exert more regulatory control biologically over the molecules in the lower tiers. Moreover, the network indicates that at least 20 of our target genes are within two nodes of Pten, representing ~5% of the molecules in the dataset (Fig. 2c). The network constructed by IPA highlights the importance of the genes experiencing changes in AS to signaling pathways that are known to be disrupted in the *Pten*^*m3m4*^ model and other models of *PTEN* mutation.

To assess the overlap in genes that have changes in AS between our two time points, we constructed a Venn diagram. We found there are 14 target genes intersected at the two time points, representing ~7–8% of each of the target gene lists (Fig. 2d; Supplementary Table 4). The lack of overlap between the two time points is surprising but consistent with the enrichment and pathway analysis results, which show distinct biological signatures with some small areas of overlap. These data suggest that the change in Pten localization and expression observed in the *Pten*^*m3m4/m3m4*^ brain has different effects on patterns of AS over the course of neurodevelopment and the early changes appear especially relevant to PTEN and ASD biology.

### Dysregulation of splicing factors in the *Pten*^*m3m4*^ cortex

**Fig. 3:**
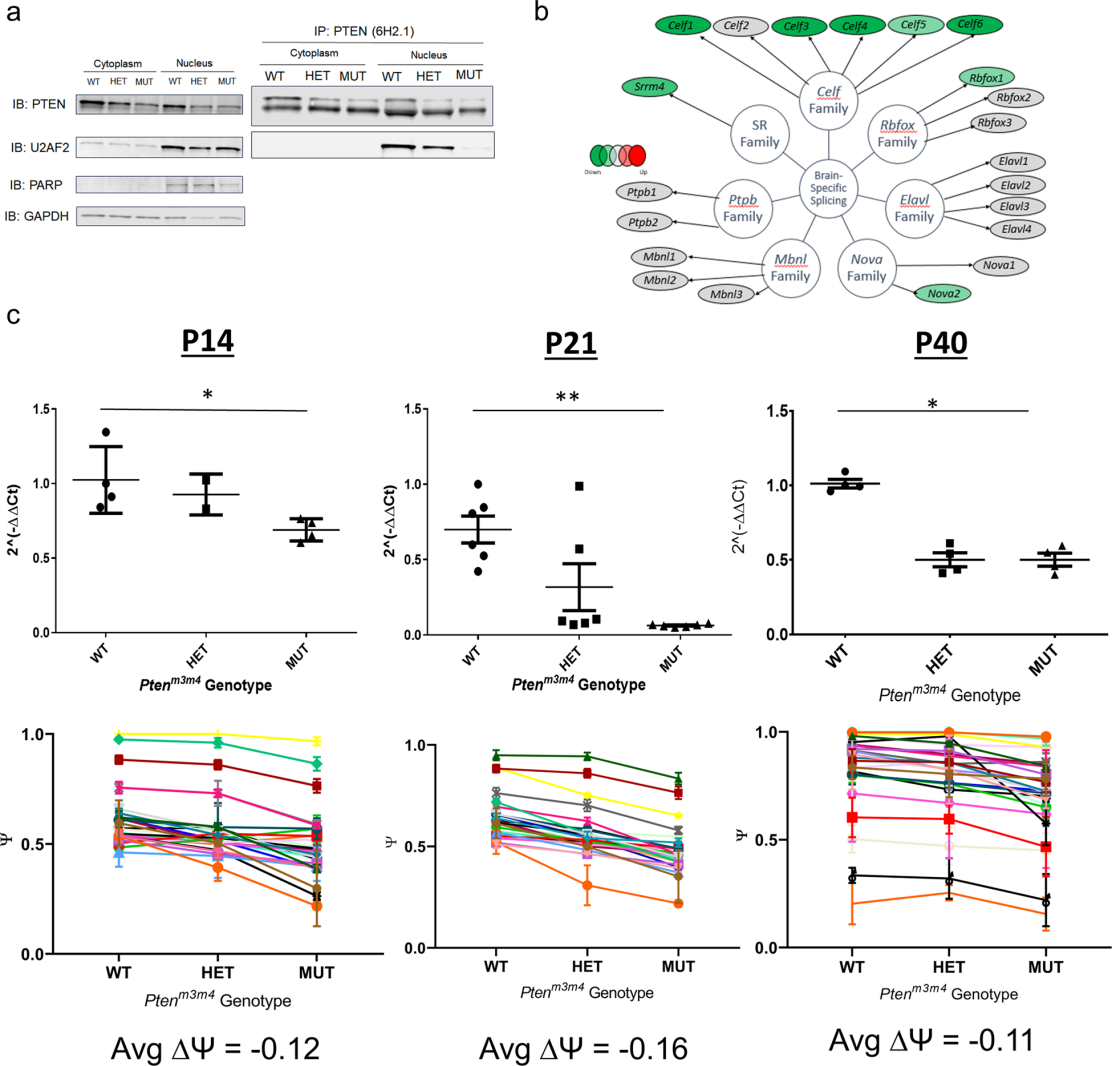
Dysregulation of splicing factors in *Pten*^*m3m4/m3m4*^ brain. **a** Co-IP for Pten and U2af2 on lysates from subcellular fractions of cortical tissue from both *Pten*^*m3m4*^ genotypes compared to wild type. **b** Schematic of P40 *Pten*^*m3m4/m3m4*^ cortical expression pattern of neural-enriched/specific splicing factors. Dark green indicates a significant decrease (log_2_fold > 1 or < −1; *p* < 0.05; *q* < 0.05) in MUT vs. WT comparison. Light green indicates significance in both MUT vs. WT and HET vs. WT comparisons. **c** *Srrm4* qRT-PCR analysis at P14 (*N* = 4), P21 (*N* = 6), and P40 (*N* = 4) with corresponding RT-PCR quantification of Srrm4-regulated microexons (*N* = 3; *p < 0.05; **p < 0.01). Color based legend of microexons in Supplementary Fig. 2.

Given that the m3m4 mutation impairs the nuclear localization of Pten^13^ and considering the observed changes in AS patterns, we examined the interaction of Pten with the known spliceosomal protein U2af2. In a co-IP experiment with nuclear/cytoplasmic fractionation, we found that U2af2 was not pulled down with Pten from nuclear fractions of cortical lysates from homozygous mutant mice in contrast to successful co-IP of Pten-U2af2 from both wild-type and heterozygous mutant cortical, nuclear lysates (Fig. 3a). This clearly demonstrates that the m3m4 mutation disrupts the Pten-U2af2 interaction, with subsequent loss exerting influences over the AS changes in the *Pten*^*m3m4*^ neural transcriptome.

We also returned to our published *Pten*^*m3m4*^ neural transcriptomic dataset^14^ to determine if there were changes in splicing factors enriched in the CNS. Of the six families of neural-enriched splicing factors^22^, we found almost complete downregulation of the *Celf* family genes as well as decreases in *Nova2*, *Rbfox1*, and *Srrm4* in the *Pten*^*m3m4/m3m4*^ cortex at 6 weeks of age (Fig. 3b). The downregulation of several brain-enriched splicing factors may be responsible, in part, for the changes in AS patterns in the *Pten*^*m3m4*^ model.

The changes in the expression of the splicing factor *Srrm4* was intriguing due to the existing ASD mouse model resulting from *Srrm4* haploinsufficiency^20,23^. Thus, we validated the change in *Srrm4* expression with qRT-PCR at three different time points: P14, P21, and P40. We found significantly decreased expression of *Srrm4* in the homozygous mutant at all three ages (*p* = 0.018, 0.0011, 0.018, respectively; Fig. 3c). Given that Srrm4 is heralded as a master regulator of microexons (exons of 3–27 nucleotides in length)^20^, we assessed the percent-spliced-in (PSI) for 27 Srrm4-regulated microexons (Fig. 3c; Supplementary Fig. 2). We found a general trend of a modest decrease in PSI (slightly above a 10% decrease on average) in the homozygous mutant cortex (Fig. 3c). This finding is consistent with the decrease in *Srrm4* expression. Furthermore, the change in Srrm4 expression and microexon inclusion may have functional consequences that contribute to ASD pathogenesis and suggest a potential shared pathophysiology between the *Pten* and *Srrm4* model of autism.

### Preference for shorter isoform of *Gfap* in *Pten*^*m3m4*^ cortex associates with astrocyte pathology

**Fig. 4:**
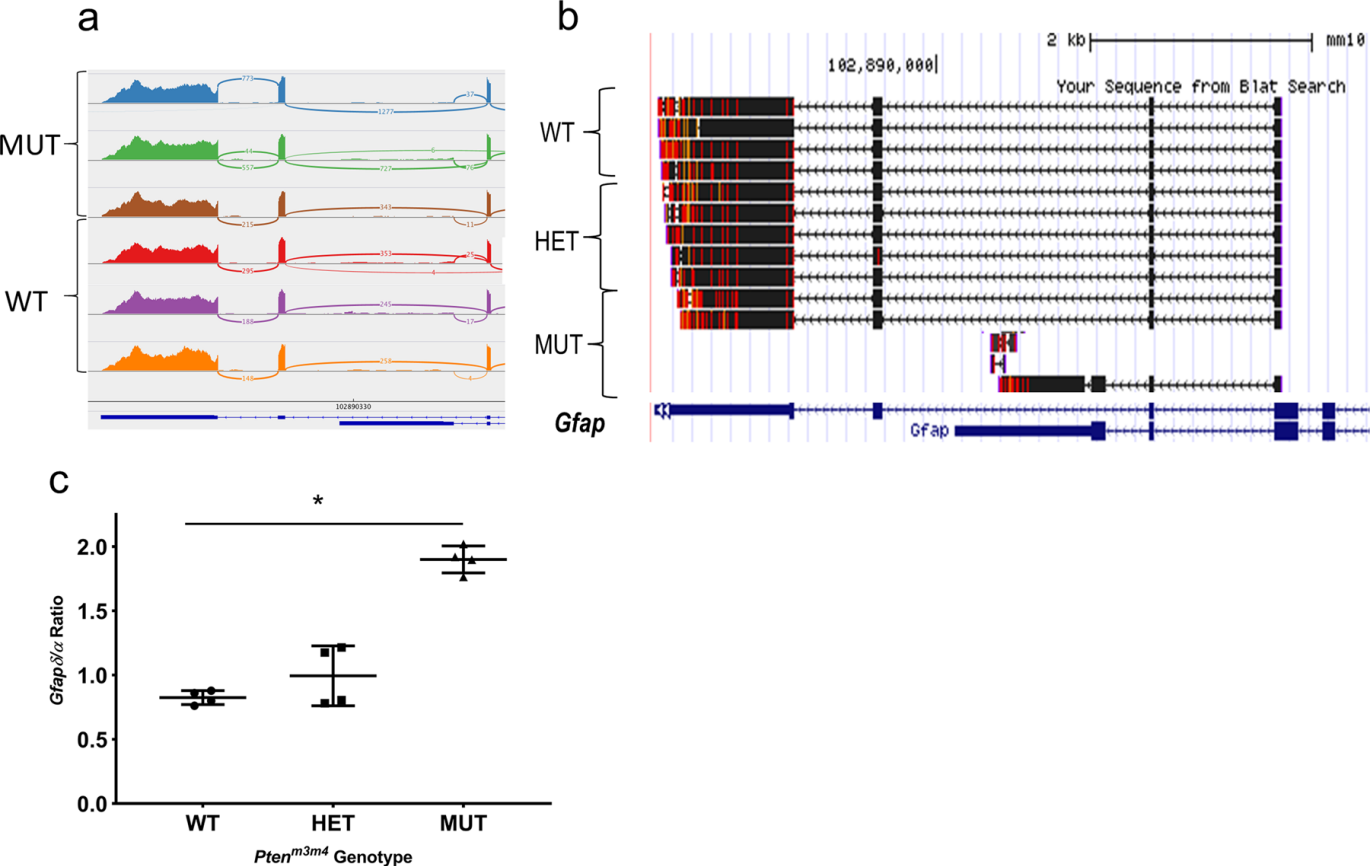
Evidence of isoform switching in Gfap, a molecular feature associated with astrocyte pathology. **a** IGV snapshot of 3′ end of the *Gfap* locus focused on gap junction reads showing evidence of Gfapδ splicing event. **b** 3′ RACE sequencing of *Gfap*, showing representative clones from both *Pten*^*m3m4*^ genotypes vs. wild type. **c** Isoform-specific qRT-PCR analysis for and Gfapδ. The ratio of the relative expression (i.e., 2^-(∆∆Ct)^) of Gfapδ to Gfapα (Gfapδ/Gfapα) is graphed (*p < 0.05).

In our initial RNA-sequencing study of the brain of the *Pten*^*m3m4*^ mouse^14^, the Cuffdiff pipeline output included an analysis on differentially expressed transcripts (Supplementary Table 5). In this previously unpublished dataset, our attention was drawn to the DE of Gfap transcripts because of its high DE and the aggressive astrocyte pathology in the mouse^13^. Subsequently, we evaluated the gap junction read data at the *Gfap* locus between homozygous mutant and wild-type mice by visual inspection using the Integrated Genome Viewer. We found that evidence of gap junction reads for the shorter isoform of *Gfap*, i.e., Gfapδ, are approximately three times more prevalent (an average of 45 vs. 15 reads) in the homozygous mutant cortex compared to reads from the wild-type cortex (*p* = 0.047; Fig. 4a).

In order to confirm this finding as looking at raw reads does not account for changes in read counts across the locus, we performed 3′ RACE sequencing on wild type, heterozygous mutant, and homozygous mutant cortices at age P40 to ascertain a general idea of the transcript population from *Gfap*. Strikingly, we could only find evidence of Gfapδ in the homozygous mutant RACE clones (Fig. 4b). Furthermore, we performed an isoform-specific qPCR analysis to selectively amplify the two major isoforms of *Gfap*, i.e., Gfapα and Gfapδ, from the cortex of all genotypes of *Pten*^*m3m4*^ mice. Consistent with our previous findings on *Gfap* expression, we found an overall increase in all isoforms of *Gfap*, but we found a clear increase, approximately twofold increase, in the prevalence of the ratio of Gfapδ to Gfapα, indicating a relative enrichment in preference for the shorter isoform (Fig. 4c). These findings confirm that despite an overall increase in the expression of Gfap, there is a switch in isoform preference in the *Pten*^*m3m4/m3m4*^ cortex relative to the wild-type and heterozygous mutant cortex. We have not demonstrated any causal relationship, but this increase in *Gfap* expression and the isoform switching occur in parallel with the most prominent glial phenotype in the *Pten*^*m3m4/m3m4*^ mouse, astrogliosis^13^.

## Discussion

Building on our previous observation that the Pten model’s neural transcriptome mimics human idiopathic autism, we now show that the m3m4 mutation can disrupt the many aspects of Pten function that presumptively participate in the regulation of AS. Our rMATS analysis found that each m3m4 allele introduces a similar disruption to the landscape of AS in the brain (roughly 200–250 ASEs), suggesting that Pten-regulated splicing is multifaceted. Although inclusion/exclusion and ΔΨ distributions were similar across event types and across pairwise genotype comparisons, in the most interesting (due to greatest phenotypic disparity) comparison, we found a significant increase in RI at 2 weeks of age in homozygous mutants compared to wild type (Fig. 1). There are 16 genes experiencing RI and 25% of these genes are related to calcium signaling, ostensibly important in the brain, especially to synaptic transmission. RI generally results in a decrease in protein expression so it is possible that the RI may contribute to the decrease in synaptic transmission that was predicted by our RNA expression data^14^. Importantly, this RI is observed at the earlier time point, and thus, may exert more developmental influence. In the few studies of AS in ASD individuals and models^4,7,20,23^, RI events have been found, but a prominent shift towards RI identified from a global comparison of splicing by event type has not been observed. It is possible that this shift toward RI is specific to PTEN-ASD, relating to some important developmental roles that Pten plays in promoting the excision of introns in the developing nervous system.

To understand more about the likely biological consequences of the changes in AS, we performed gene enrichment and pathway analysis on the genes with ASEs from the MUT vs. WT comparison. We identified several pathways related to PTEN function or metabolism of PTEN substrates, especially at the P14 time point, and significantly enriched biological process networks consistent with phenotypes observed in *Pten*^*m3m4/m3m4*^ mice, such as changes in brain morphology and development (Fig. 2)^13^. Although it is difficult to disentangle whether the ASEs drive or simply accompany ASD-related changes in the transcriptome, the existence of the correlation itself is relevant to the pathophysiology of PTEN-ASD. Moreover, elucidating these ASEs highlights a burgeoning role for Pten in the process of AS, which heretofore has been underappreciated.

What is clearer than whether ASEs are, in part, a cause or an effect or aspects of PTEN-ASD is the role of Pten in the regulation of AS. Previous work has shown that PTEN interacts with several splicing factors, notably many members of the hnRNP splicing factor family^18,24^. Moreover, it is well understood that several kinase-activated signaling cascades, of which PTEN is a critical antagonist, regulate the phosphorylation status of splicing factors, especially those belonging to the SR-protein family^25^. These studies implicate PTEN as an underrecognized regulatory node in the complex network of factors that regulate splicing mechanisms. Our study expands on this work by demonstrating a clear dysregulation of neural-enriched splicing factors, especially *Celf* family genes (Fig. 3). The almost global decrease in *Celf* family gene expression in the mutant cortex was an important observation as *Celf* knockout models have neurodevelopmental or neurological phenotypes and *CELF* variants have been associated with ASD^26^. Moreover, the role of Pten in regulating *Celf* genes has recently been supported by an adjacent observation in HCT116 cells, where *CELF6* knockout increased PTEN expression^27^. In light of our finding in the *Pten*^*m3m4*^ model, these results may together suggest possible reciprocal regulation between *PTEN* and *CELF* family genes, where PTEN drives *CELF* expression and that cells attempt to compensate for *CELF* expression loss by upregulating *PTEN* expression.

An additional aspect of PTEN’s regulation of AS is the ability of PTEN to recruit U2AF2 to the spliceosome^18^. Our study is an important validation of the PTEN-U2AF2 interaction. We find that this interaction is conserved in mice and present in the brain not just HEK293 cells. Moreover, we show that a mutation that decreases the localization of Pten to the nucleus disrupts this interaction (Fig. 3). Although Shen et al identified a similar number of ASEs, their set of ASEs showed little to no overlap with our ASEs (data not shown), which may be explained by the differences in models (i.e., a small hairpin knockout in human immortalized cells vs. the brain of a germline knockin mouse). The differences between these two datasets may also be explained by the change in neural-enriched splicing factor expression and may suggest the importance of accurate spatial relationships among cells and at least, partial non-cell autonomy. Furthermore, understanding the source of the discrepancy between these two models may be illuminating in attempting to tease out the clinical heterogeneity associated with different *PTEN* genotypes. These discrepancies, in fact, may tease out the differences in AS defects downstream of incorrect cellular localization of Pten (m3m4) and downstream of haploinsufficiency. The splicing-related phenotype observed in the *PTEN* knockout HEK293 cells was somewhat more related to PHTS neoplasia phenotypes (i.e., golgi extension and increased secretion) than neurodevelopmental phenotypes. The comparison of these two studies hints that Pten disruption may have tissue-specific effects on the landscape of splicing, meaning the events subject to regulation via Pten function in the CNS may differ entirely than those in peripheral tissues. Thus, the differential effects on the spliceosome due to *PTEN* loss and *Pten*^*m3m4*^ may be important to understanding how different *PTEN* genotypes can lead to different clinical phenotypes.

A deeper understanding of how specific disruptions of PTEN function affect splicing regulation may help tease out the phenotypic complexity associated with germline *PTEN* mutations. It is possible that specific mutations induce splicing patterns that contribute to or maintain certain PHTS phenotypes. For instance, the m3m4 mutation in *Pten* presents similarly to clinically observed mutations (e.g., *PTEN* D252G) in terms of the pattern of Pten expression, cytoplasmic-predominance, which is an expression pattern that is associated with autism rather than neoplasia^13–17^. All mutations that restrict PTEN from the nucleus will likely disrupt the PTEN-U2AF2 interaction in neurons and will subsequently share at least some similar splicing changes.

Another crucial finding related to splicing factor dysregulation in the *Pten*^*m3m4*^ model was the decrease in *Srrm4* expression at three different developmental time points and the corresponding decrease in Srrm4-regulated microexon inclusion (Fig. 3b, c). This finding was phenotypically similar to *nSR100*^*+/Δ7–8*^ mouse with *Srrm4* haploinsufficency^20^, though the decrease in microexon inclusion was less dramatic. The *nSR100*^*+/Δ7–8*^ mouse displays autism-like phenotypes, such as sexually dimorphic social impairment and impaired synaptic transmission^20^. These phenotypes share significant similarity with the *Pten*^*m3m4*^ mouse^13–15^. The similarity of the AS landscape of two separate genetic models of autism suggests potentially convergent biological mechanisms involved in ASD initiation and maintenance. At the very least, the shared biology represents an important avenue of future study.

Another change in the transcriptional landscape with a clear molecular correlate in the *Pten*^*m3m4*^ model was the the isoform switching in *Gfap*, where Gfapδ, the major shorter isoform, is increased relative to Gfapα, the major longer isoform (Fig. 4). Although *Pten*^*m3m4/m3m4*^ mice show a global increase in total Gfap expression^13,14^, there is also a dramatic change in the ratio of Gfapδ and Gfapα. Importantly, Gfapδ is expressed by neurogenic astrocytes especially from the subpial layer and subventricular zone and exerts significant influence over astrocyte size, morphology, and motility^28–30^. Gfapδ immunoreactivity is common to astrocytoma^31^. In the *Pten*^*m3m4/m3m4*^ model, the most prominent, dramatic glial phenotype is astrogliosis. The Gfap isoform switching is thus a correlate of the observed astrocyte phenotype subsequent to Pten disruption. However, it is unclear if any causal relationship exists between the astrocyte pathology and the isoform switching. Future research is needed to parse out the mechanisms by which Pten regulates Gfap isoform switching and the nature of its association with the astrocyte pathology.

Together, these data illustrate the importance of Pten to the landscape of AS in the brain and to the regulation of AS, presumptively leading to ASD (Supplementary Fig. 3). Pten disruption that affects both nuclear localization and protein stability is able to induce changes in AS that affect a couple hundred genes, genes that are important to PTEN and neuron biology. Moreover, there is an important shift toward RI in the mutant brain, especially earlier in development, that likely affects the expression of the genes exhibiting RI. The fact this shift is more prominent early on suggests it may exert more developmental influence over the model’s phenotypes. The multitude of levels at which Pten engages with players important to the regulation of AS suggests a complex and subtle yet critical role for Pten. Use of a single model and single organism provides limited insight into how Pten dysfunction specifically influences ASEs and how these changes in the landscape of AS affect ASD phenotypes, but this study demonstrates a clear regulatory role for Pten in AS mechanisms in the brain and suggests that the changes in the AS landscape are relevant to PTEN-ASD phenotypes.

## Supplementary information

Supplementary Information
