# Supplementary material for: Alternative splicing landscape of the neural transcriptome in a cytoplasmic-predominant Pten expression murine model of autism-like Behavior
Source: Transl Psychiatry. 2020 Nov 6;10:380. doi: 10.1038/s41398-020-01068-x (PMC7648763; doi:10.1038/s41398-020-01068-x)
Supplement: Supplementary file 1 — Supplementary Information [file 41398_2020_1068_MOESM1_ESM.docx]

**Supplementary Information**

**Alternative Splicing Landscape of the Neural Transcriptome in a Cytoplasmic-Predominant Pten Expression Murine Model of Autism-like Behavior**

Stetson Thacker BS, Marilyn Sefyi MS, Charis Eng MD PhD

**Table 1.** Primer details for microexon alternative splicing analysis

| **Primer** | **Coordinates (mm9 assembly)** | **Sequence** | **PCR_SIZE** | **Tm** |
| --- | --- | --- | --- | --- |
| **ArvcfF** | chr16:18,400,178-18,401,731 | CAGCGACGGAGGAAGGATGA | 114-132 | 61.4 |
| **ArvcfR** |  | CTCCGTTCGTTTAGGCAGGTC | 114-132 | 60.1 |
| **Dnm2F** | chr9:21,294,187-21,295,870 | AAGAGGGCCATACCCAATCAG | 86-98 | 58.5 |
| **Dnm2R** |  | TACTCCTTGGAGCCACCCTTC | 86-98 | 60.9 |
| **Dync2h1F** | chr9:7,044,330-7,049,192 | CTGGAAAAGTGAGGGCCTTCC | 118-139 | 60.5 |
| **Dync2h1R** |  | TAGCCACTCTGTTGCCTGAGA | 118-139 | 59.6 |
| **Itsn1F** | chr16:91,841,734-91,849,214 | CGATGAGATCACCATCCAGCC | 102-117 | 59.8 |
| **Itsn1R** |  | CATCCCGTCTTCCCTTTCAGC | 102-117 | 60.4 |
| **NbeaF** | chr3:55,796,256-55,804,562 | CTCAGCAGCTACATCGCCAAC | 108-117 | 60.4 |
| **NbeaR** |  | TCAACCATTGCCATGAGTCGG | 108-117 | 59.4 |
| **Ppfia2F** | chr10:106,306,935-106,330,569 | CTGGGCTTAGGCAAACTTGGA | 115-124 | 59.4 |
| **Ppfia2R** |  | GTCCATCCCACTGGGCAAAAG | 115-124 | 60.8 |
| **PtprdF** | chr4:75,696,231-75,705,733 | CCTGTCCTTGCAGTGGTGTTT | 92-104 | 59.3 |
| **PtprdR** |  | CTGCTCTTTCTGGACTCCGAC | 92-104 | 59.6 |
| **Pus7F** | chr5:23,268,181-23,274,730 | GACAGAGGATAGAGAGGGGCG | 108-126 | 61.2 |
| **Pus7R** |  | TAACTTCCCCGGGATTTTGGC | 108-126 | 59.9 |
| **Rapgef6F** | chr11:54,462,684-54,470,988 | ATCCAGCAGCCCAGATCTCTT | 113-128 | 59.8 |
| **Rapgef6R** |  | AGCAACTTTGTTGATCTGCTTTGA | 113-128 | 56.4 |
| **Shank1F** | chr7:51,599,028-51,599,944 | CATCTCCCTGCGTTCCAAGTC | 111-135 | 60.2 |
| **Shank1R** |  | GCCATCTGATACACAGTCCGC | 111-135 | 60.1 |
| **Slit2F** | chr5:48,650,783-48,666,914 | TACACATGTCTTTGCCCACCG | 119-146 | 59.2 |
| **Slit2R** |  | CCCTTTGGAGTCAGGATGCAC | 119-146 | 60.2 |
| **Synj1F** | complex | CCAGAAGGATTCTTCCCAGAC | 132-171-179-210-258 | 56.8 |
| **Synj1R** |  | CTTGAGGGGAAGGCTGATTAC | 132-171-179-210-258 | 57.1 |
| **Vav2F** | chr2:27,154,894-27,157,630 | ATCTACGACTGTGTCCCGTGT | 117-132 | 59 |
| **Vav2R** |  | GCAGCTTCTCTTGTCGTCCTC | 117-132 | 60.2 |
| **Zmynd8F** | chr2:165,677,500-165,701,275 | GAGGTGGTGGAGGGAATGGAT | 106-118 | 60.9 |
| **Zmynd8R** |  | CATTGGAGGAATGTGGAGGGC | 106-118 | 60.5 |
| **Vps13cF** | complex | TGCCGAATTGAAGAAGCCCTT | 133-196-262 | 59 |
| **Vps13cR** |  | TGTGAATGTCTGTAATTTTCACTTGA | 133-196-262 | 53.5 |
| **Clasp2** | chr9:113,785,362-113,789,304 | TGGAGGAGGCAGTAGCTGATG | 117-141 | 60.6 |
| **Clasp2** |  | AGCGTTCTGAACACGCACTAG | 117-141 | 58.9 |
| **Dctn1** | chr6:83,145,935-83,147,618 | CATCGCTACCCTGGTCTCTGG | 106-121 | 61.6 |
| **Dctn1** |  | CTGAAGCAGCAGTGGGGAGTC | 106-121 | 62.6 |
| **Pls3F** | chrX:73,044,734-73,045,983 | CTGGAAAACTCAGGCTGGCAA | 123-150 | 59.8 |
| **Pls3R** |  | TATCCGTGGCTCCCCTTCTTT | 123-150 | 60.1 |
| **Dock4** | chr12:41,516,663-41,521,450 | CGGAGGGCAAAGGTGATGAAA | 117-144 | 59.7 |
| **Dock4** |  | GCCGAGTGACAGTGGCAATTA | 117-144 | 59.4 |
| **Kif1b** | chr4:148,637,724-148,640,426 | AAAGTCATTTCGGCCTTGGCA | 102-120 | 59.3 |
| **Kif1b** |  | GAGATTTTCTCGAAGGAGCCAAGT | 102-120 | 58.7 |
| **Slc38a10** | chr11:119,967,748-119,970,850 | GGAGAAGAAGGAGGCTGAGCA | 118-142 | 61.2 |
| **Slc38a10** |  | CTGCTGCTCTTGGATCACCTG | 118-142 | 59.9 |
| **Dtna** | chr18:23,755,935-23,761,175 | TGCTTCAAGCCGTGAACCTTT | 99-108 | 59.1 |
| **Dtna** |  | ATGCTGGTTACAGGTCTCGGA | 99-108 | 59.5 |
| **Sh3glb1** | chr3:144,360,342-144,368,569 | TGGATGCTGCAAAAACAAGACT | 115-139 | 56.5 |
| **Sh3glb1** |  | CGGGTAATCTCTGCCTGACGA | 115-139 | 60.8 |
| **Mta1F** | chr12:114,371,752-114,374,039 | GCAACATGAAGAAGCGCCTCT | 68-80 | 60 |
| **Mta1R** |  | CATGTGCCTGGTCTGTCCATG | 68-80 | 59.9 |
| **Zfyve27F** | chr19:42,258,608-42,260,439 | AGTGTGGAGGAAGCTGAGGAG | 118-139 | 60.4 |
| **Zfyve27R** |  | ATCCATTGTCCTGCAGGGTCA | 118-139 | 60.2 |
| **Lass6F** | chr2:68,943,060-68,952,339 | TCCTGGTGGGTTTTTAACCTGCT | 208-232 | 60.4 |
| **Lass6R** |  | TGGTTCCGTTGGTGGTTGTTGAAG | 208-232 | 61.7 |
| **Mef2dF** | chr3:87,965,113-87,967,009 | ACAAAGTCATCCCTGCCAAGTCTC | 209-230 | 60.9 |
| **Mef2dR** |  | GAGTAAACTTGGTGTTGCCACGGA | 209-230 | 61.4 |

**Table 2.** Homozygous mutant versus wildtype rMATS 4.0.2 summarized results

| **Age** | **Splice Type** | **Inclusion Events** | **Exclusion Events** | **Total Events** | **Inclusion dPSI** | **Exclusion dPSI** | **Total dPSI** |
| --- | --- | --- | --- | --- | --- | --- | --- |
| P14 | SE | 90 | 69 | 159 | 0.36 | -0.36 | 0.05 |
| P14 | A3SS | 10 | 13 | 23 | 0.4 | -0.38 | -0.04 |
| P14 | A5SS | 7 | 11 | 18 | 0.38 | -0.38 | -0.08 |
| P14 | RI | 16 | 4 | 20 | 0.41 | -0.36 | 0.26 |
| P40 | SE | 72 | 71 | 141 | 0.32 | -0.33 | -0.01 |
| P40 | A3SS | 8 | 7 | 15 | 0.39 | -0.42 | 0.01 |
| P40 | A5SS | 6 | 15 | 21 | 0.38 | -0.35 | -0.14 |
| P40 | RI | 11 | 6 | 17 | 0.46 | -0.51 | 0.12 |

**Table 3.** Summary of mutually exclusive splicing event for all *Pten^m3m4^* genotype comparisons.

| **Age** | **Comparison** | **Events with Exon A Favored** | **Events with Exon B Favored** | **Average Delta PSI** |
| --- | --- | --- | --- | --- |
| P14 | MUT vs WT | 13 | 5 | (A) 32.6%  (B) 41.9% |
|  | HET vs WT | 11 | 8 | (A) 19.7%  (B) 27.1% |
|  | MUT vs HET | 5 | 5 | (A) 46.4%  (B) 27.5% |
| P40 | MUT vs WT | 5 | 6 | (A) 33.2%  (B) 46.3% |
|  | HET vs WT | 13 | 17 | (A) 20.8%  (B) 26.6% |
|  | MUT vs HET | 15 | 13 | (A) 26.4%  (B) 28.9% |


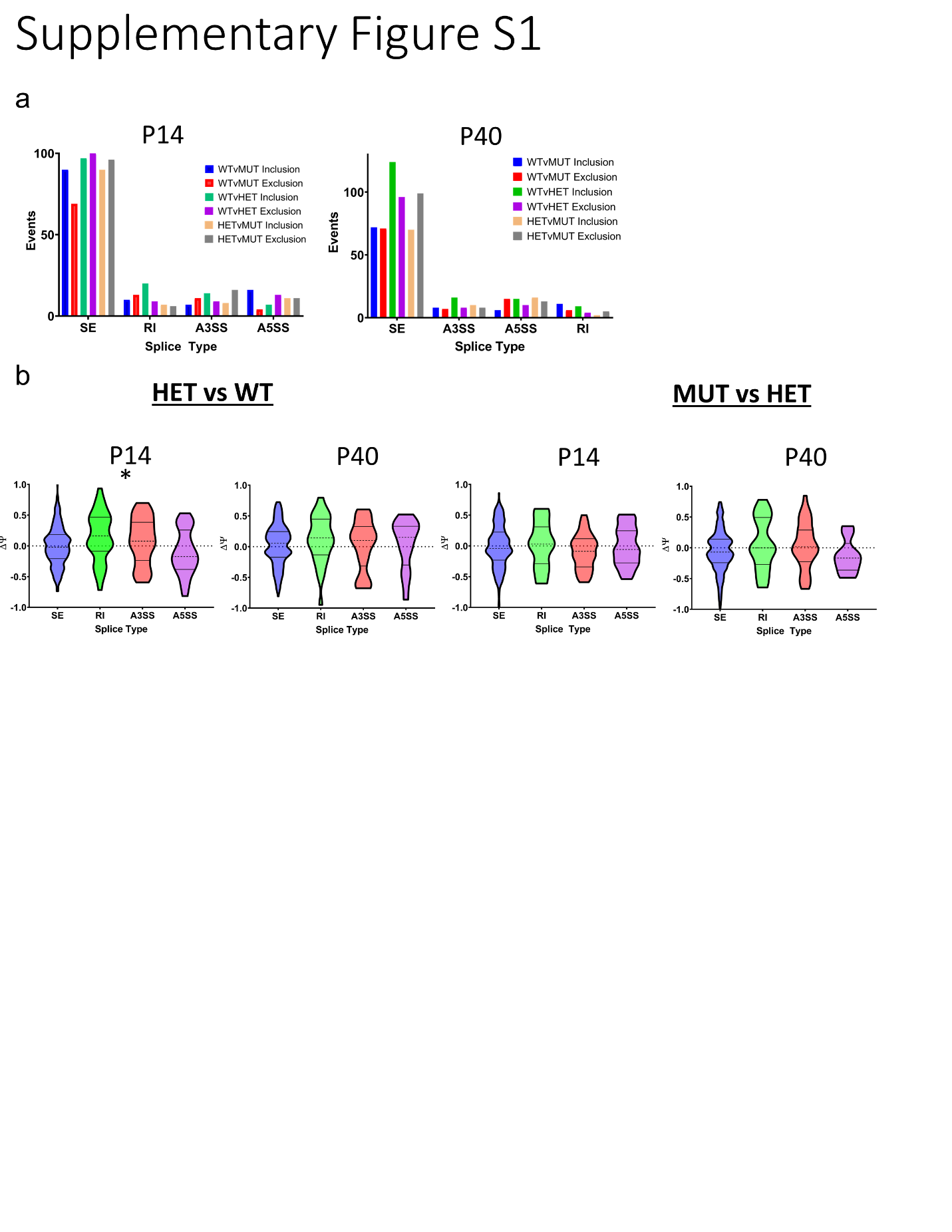


**Figure 1.** Summary of rMATS analysis of *Pten^m3m4^* neural transcriptome (N = 3 for each genotype at each time point). **a** Overview of inclusion and exclusion events for all genotype comparisons at both P14 and P40. **b** Violin plots of distribution of delta PSI distributions by event type for HET vs WT and MUT vs HET comparisons. Kruskal-Wallis identified a significant difference among group in HET vs WT P14 (p < 0.05) comparison but correction during post-hoc testing found none of the pairwise comparison reached significance. Significance, false discovery, and delta PSI filters used for these data.

**Table 4.** List of genes with significant ASEs shared by both P14 and P40 time points.

| **Gene Name** | **Gene Description** |
| --- | --- |
| *Slc16a7* | solute carrier family 16 (monocarboxylic acid transporters), member 7 |
| *Ankrd45* | ankyrin repeat domain 45 |
| *Ing4* | inhibitor of growth family, member 4 |
| *Cdkl3* | cyclin-dependent kinase-like 3 |
| *Lsm7* | LSM7 homolog, U6 small nuclear RNA and mRNA degradation associated |
| *Letm2* | leucine zipper-EF-hand containing transmembrane protein 2 |
| *Zfp862-ps* | zinc finger protein 862, pseudogene |
| *Acbd5* | acyl-Coenzyme A binding domain containing 5 |
| *Mecom* | MDS1 and EVI1 complex locus |
| *Ciz1* | CDKN1A interacting zinc finger protein 1 |
| *Junos* | jun proto-oncogene, opposite strand |
| *Ptk2* | PTK2 protein tyrosine kinase 2 |
| *Lekr1* | leucine, glutamate and lysine rich 1 |
| *Arfgap1* | ADP-ribosylation factor GTPase activating protein 1 |

**
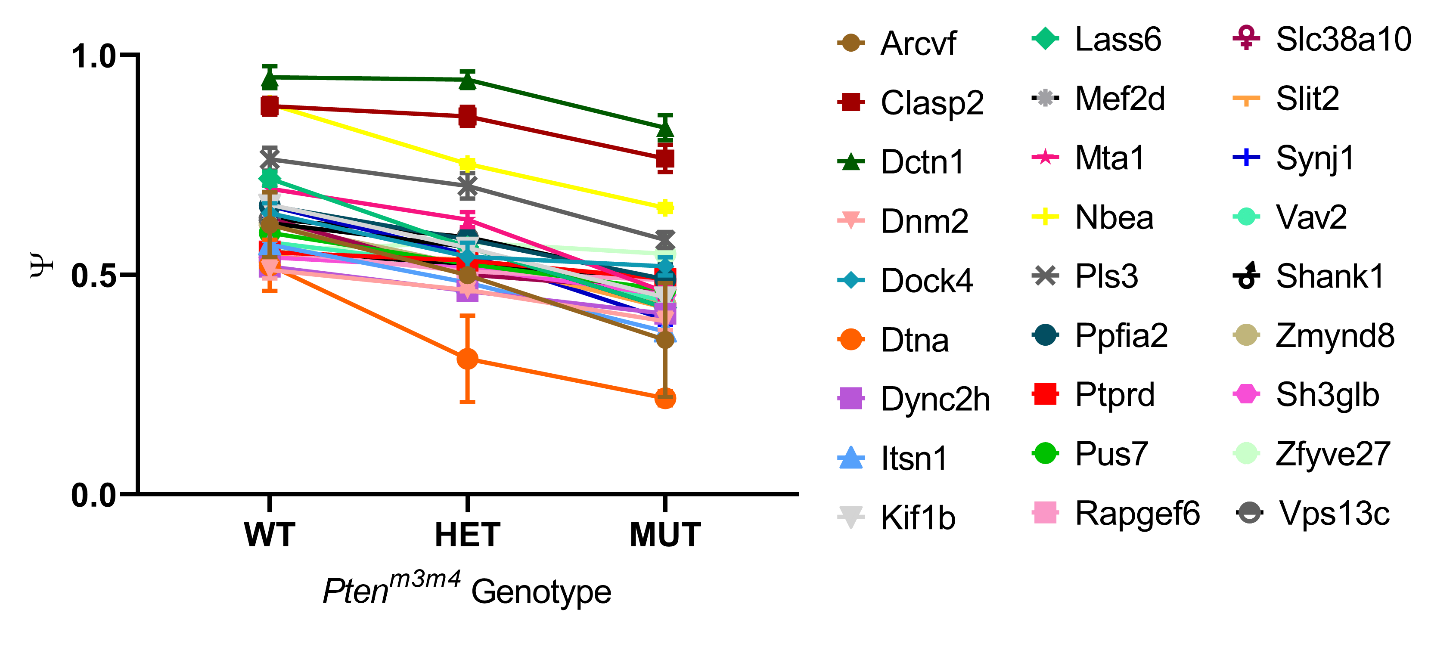
**

**Supplementary Figure 2.** Color legend for Srrm4-regulated microexons.

**Table 5.** List of differentially expressed transcripts in *Pten^m3m4/m3m4^* cortex relative to wildtype cortex, showing top 10 most upregulated and top 5 most downregulated transcripts.

| **Gene ID** | | **Isoform** | **WT (FPKM)** | **MUT (FPKM)** | **Fold Change** | **P-value** |
| --- | --- | --- | --- | --- | --- | --- |
| *Rn45s* | NR_046233 | | 264.52 | 1305.95 | 4.93 | 5.00E-05 |
| *Gsn* | NM_146120 | | 30.56 | 83.50 | 2.73 | 5.00E-05 |
| *Ugt8a* | NM_011674 | | 41.66 | 109.86 | 2.64 | 5.00E-05 |
| *Gfap* | NM_010277 | | 37.00 | 93.60 | 2.53 | 5.00E-05 |
| *Plp1* | NM_011123 | | 745.86 | 1798.79 | 2.41 | 5.00E-05 |
| *C1qa* | NM_007572 | | 35.24 | 84.48 | 2.40 | 5.00E-05 |
| *Mog* | NM_010814 | | 50.47 | 119.67 | 2.37 | 5.00E-05 |
| *Igfb2* | NM_008342 | | 24.19 | 54.90 | 2.27 | 5.00E-05 |
| *Cnp* | NM_001146318 | | 98.29 | 222.31 | 2.26 | 5.00E-05 |
| *Cld11* | NM_008770 | | 126.81 | 272.70 | 2.15 | 5.00E-05 |
| *Arpp21* | NM_028755 | | 65.26 | 41.59 | -1.57 | 1.45E-03 |
| *Rasd2* | NM_029182 | | 32.57 | 20.53 | -1.59 | 5.00E-05 |
| *Lmo3* | NM_207222 | | 33.54 | 20.51 | -1.64 | 5.00E-05 |
| *Ttr* | NM_013697 | | 120.17 | 24.43 | -4.92 | 5.00E-05 |
| *Egr1* | NM_007913 | | 42.12 | 25.43 | -1.66 | 5.00E-05 |


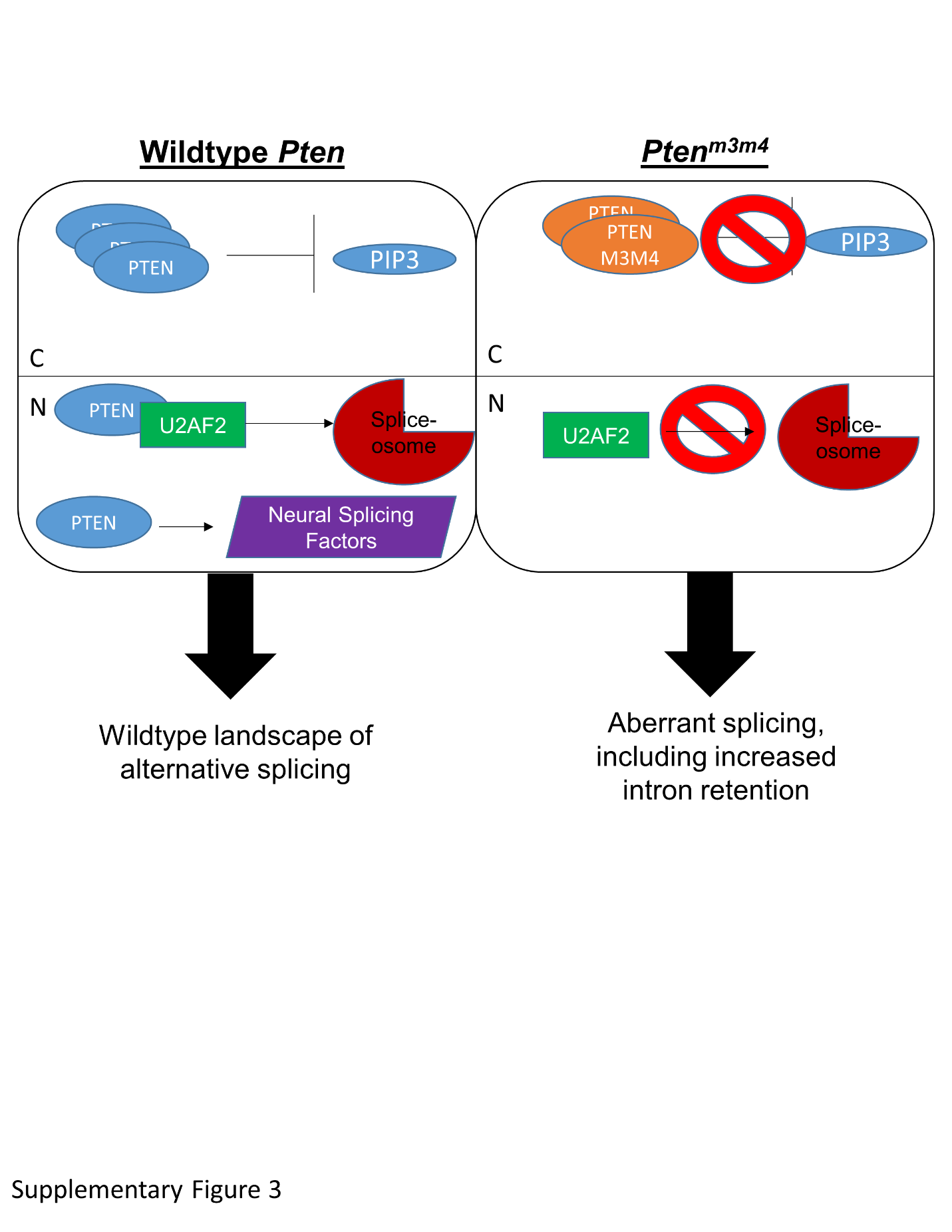


**Supplementary Figure 3.** Graphical schematic of Pten’s regulation of alternative splicing. Left panel: wildtype scenario where Pten negatively regulates Pi3k signaling in the cytoplasm, recruits U2af2 to the spliceosome in the nucleus, and promotes the expression of neural-enriched splicing factors. Right panel: m3m4 mutant scenario where Pten cannot adequately regulate Pi3k signaling in the cytoplasm due to instability (lower steady-state levels), cannot recruit U2af2 to the spliceosome because Pten is not in the nucleus at sufficient levels, and cannot promote expression of neural-enriched splicing factors.
